# First Impression Formation Based on Valenced Self-Disclosure in Social Media Profiles

**DOI:** 10.3389/fpsyg.2021.656365

**Published:** 2021-06-18

**Authors:** Yuren Qin, Hichang Cho, Pengxiang Li, Lianshan Zhang

**Affiliations:** ^1^Department of Communications and New Media, National University of Singapore, Singapore, Singapore; ^2^School of Media and Communication, Shenzhen University, Shenzhen, China

**Keywords:** online self-disclosure valence, first impression, perceived homophily, trustworthiness, likability

## Abstract

This study aims to understand how the valence of self-disclosure (operationalized as the dominantly positive vs. balanced vs. dominantly negative social media posts of a future collaborator) influences first impression formation on social media. We also focus on trustworthiness as a mediator and perceived homophily as a moderator to specify the underlying mechanisms through which self-disclosure valence affects first impression formation. The results from an online experiment (*N* = 204) suggest that self-disclosure valence has a significant effect on perceived trustworthiness and likability when individuals evaluate an unknown future collaborator using the social media profile. Trustworthiness mediates the effect of self-disclosure valence on likability when the individuals feel that they are dissimilar or even slightly similar to strangers. At that time, individuals tend to seek cues from both self-disclosure valence and perceived homophily to form the trustworthiness perception, and the influence of self-disclosure depends on the level of perceived homophily.

## Introduction

Impression formation is “a process by which an organized overall impression emerges in which single traits receive specific meanings” (Bierhoff and Klein, 1989, p. 2). First impression is a salient topic in the domain of impression information because the first impression can lead to biased judgments of subsequent information in offline contexts (Asch, 1946) and online environments (Walther, 1993). Social media actively reconfigures the ways in which individuals socialize with other individuals (Orben and Dunbar, 2017) because users can passively consume information provided by other social media users, which is quite different from directed and reciprocated offline interaction. A common example of passive consumption in our daily life is viewing the posts of other social media users without interacting with them (Orben and Dunbar, 2017). In online settings, individuals frequently encounter strangers, and they make inferences based on the profiles of these strangers without any prior interaction (Bacev-Giles and Haji, 2017). Moreover, it is clear that individuals search for strangers or build initial contacts with strangers *via* social media platforms, such as LinkedIn or Facebook. As a result, it is crucial to understand how individuals make initial impression judgments, especially considering that it is becoming more widespread and convenient to get acquainted with a stranger based on their self-disclosure information on their social media profiles.

An important dimension of personal self-disclosure is valence (Gilbert and Horenstein, 1975). In terms of self-disclosure, valence refers to the extent to which “the information shared is positive, neutral, or negative” (Orben and Dunbar, 2017, p. 490). The positivity or negativity of the information disclosed has a significant influence on impression formation. Usually, positive (vs. negative) self-disclosure has a favorable (vs. negative) effect on impression formation, while in some cases, negative self-disclosure is preferred and leads to unexpected positive influence (Runge and Archer, 1981). For example, individuals who disclose negative information may be perceived as honest, which may allow them to obtain further favorable impression judgments (Robinson et al., 1995). On the contrary, individuals who disclose too much positive information may be considered dishonest, which will in turn influence the evaluation of their impressions. As such, the mixed findings of the influence of information valence on impression formation (e.g., Robinson et al., 1995; Orben and Dunbar, 2017) have led to a demand for further exploration.

Previous studies have suggested that the level of trustworthiness of disclosure information plays a crucial role in the effect of valence on interpersonal perception (Runge and Archer, 1981; Robinson et al., 1995). Moreover, the role of trustworthiness could be more significant in passive online consumption. In this social context, the only reference available for a perceiver is the stranger's self-disclosure information on their online profile. However, online profiles tend to be overly curated and managed by users for self-promotion, self-enhancement, and impression management (Toma and Hancock, 2011), making the trustworthiness of the disclosed information (e.g., valence) questionable.

Furthermore, the context of this study indicates a high level of uncertainty because the target is a stranger, and the only reference is their questionable self-disclosure information. With higher levels of uncertainty, individuals tend to look for more signals to “fill the gaps” when making interpersonal judgments (Spence, 1974). Quick interpersonal judgments are likely to be based on trust, and individuals tend to use social categories (e.g., homophily) when there are no other reliable social contextual cues (Robert et al., 2009). Therefore, this study focuses on two additional factors—perceived trustworthiness as a mediator and perceived homophily as a moderator—both of which are crucial variables that influence uncertainty reduction in initial encounters (Prisbell and Andersen, 1980; Robinson et al., 1995; Wout and Sanfey, 2007).

In sum, this study aims to test a research model that specifies the underlying mechanisms through which self-disclosure valence influences a perceiver's first impression formation by focusing on the mediating role of trustworthiness and the moderating role of perceived homophily. We tested our research model in the context of WeChat, which is a popular social media platform in China that is similar to Facebook and has more than one billion monthly active users (Tencent, 2018). Compared to Facebook, where strangers can view both self-generated information and other-generated information, WeChat provides an ideal platform to test self-disclosure valence on user profiles because strangers can only see self-generated information. Given that most online profile studies are limited to Facebook (Shu et al., 2017), conducting a study with WeChat could provide insights into self-presentation and impression formation on social media in different contexts.

This study aims to make a theoretical contribution by exploring the first impression formation of different forms of valenced self-disclosure in online profiles. It could provide robust evidence for further exploration of the effect that self-disclosure valence has on interpersonal perceptions. Previous studies have had mixed findings in this regard. Moreover, this study may contribute to a revelation regarding the underlying mechanism of valence effect because it is not restricted to testing the direct effect of self-disclosure valence. We also specify the mediation effect of perceived trustworthiness and investigate the moderation effect of perceived homophily.

## Literature Review

### Self-Disclosure Valence and Interpersonal Perception

Self-disclosure is defined as “any message about the self that a person communicates to another” (Wheeless and Grotz, 1976, p. 338). Kim and Dindia (2011) demonstrated the powerful influence that computer-mediated communication (CMC) has on self-disclosure and extended the definition of online self-disclosure by considering the traditional verbally revealing self as well as self-related pictures and links that are posted online. Considering the rapid changes in the affordances of online settings, we operationalized self-disclosure on social media as any self-generated information (conveyed verbally or nonverbally) that provides cues that allow receivers to learn more about the profile owner. Valence is a key element of self-disclosure that varies in its degree of positive vs. negative information (Wheeless and Grotz, 1976). Thus, in this study, we categorized self-disclosure valence into positive, neutral (balance of positive and negative), and negative conditions. To reflect self-disclosure in real life, we also tried a more refined look into self-disclosure valence by further splitting the positive self-disclosure condition into all positive self-disclosure and mostly positive self-disclosure (similar in the negative self-disclosure condition).

Previous literature has shown that valence (positive vs. negative) has a significant effect on individual perceptions of others. Generally, disclosing positive information is more likely to form a favorable impression (Gilbert and Horenstein, 1975; Goodmon et al., 2015; Rains and Brunner, 2015). According to social exchange theory, it may be rewarding to build a relationship with a discloser who conveys positive information in an initial interaction (Gilbert and Horenstein, 1975). Goodmon et al. (2015) also found that participants had a lower likability judgment for those who disclosed negative information about themselves (e.g., being responsible for a negative incident). Consistent with these studies, Rains and Brunner (2015) argued that individuals with positive personal information achieve more interpersonal liking, especially when the relationship is not close. The findings are in line with social penetration theory (Altman and Taylor, 1973), which suggests that people are motivated to disclose positive personal information and conceal negative aspects of themselves to make others perceive them as rewarding partners. Hence, we predicted that users with dominantly positive self-disclosure in their WeChat profiles would gain the highest level of likability.

H1: Dominantly positive online self-disclosure attains a higher level of likability compared to neutral self-disclosure, followed by negative self-disclosure.

### The Mediating Role of Perceived Trustworthiness

While it is intuitively logical to assume that positive self-disclosure leads to higher likability levels (e.g., Goodmon et al., 2015; Rains and Brunner, 2015), it is also possible that the relationship could be changed when the effect is mediated by perceived trustworthiness. Trustworthiness perception is an essential antecedent of interpersonal trust (Lau et al., 2008), and it comprises information that is used to judge whether others are trustful or distrustful and whether they are worthy of being approached or should be avoided (Wout and Sanfey, 2007).

In online settings, an interesting phenomenon involving the hyperpersonal model is online deceptive self-presentation (Walther et al., 2015). Text-based CMC allows users to selectively present their ideal selves; they can convey only those cues that they desire to share. This is a prominent trend because editable profiles allow users to rewrite and revise their disclosure information to continually make themselves more appealing (Toma and Hancock, 2011). However, perceivers may consider these positive cues unreliable and untrustworthy due to the ease of editing. Thus, the perceived trustworthiness of online self-disclosure is crucial for the influence of disclosure cues, especially with online profiles.

Previous results regarding the effect of valence on trustworthiness have varied (e.g., Runge and Archer, 1981; Robinson et al., 1995; Miller et al., 2013). Runge and Archer (1981) found that a confederate who disclosed positive personal information attained greater positive judgments of trustworthiness compared to one who disclosed negative personal information. Similarly, Miller et al. (2013) argued that negative self-disclosure is negatively associated with perceived trustworthiness, possibly because this type of personal information reveals character weaknesses and personal failures. However, Robinson et al. (1995) notably discovered the following: individuals who presented themselves in a balanced way were rated the most honest; individuals who presented themselves in a negative way were rated less honest, and individuals who presented themselves in an extremely positive way were rated the least honest. Self-disclosure that is too positive is regarded as a form of extreme self-enhancement, which seems to run counter to an individual's expectations during initial interactions, and it further urges them to consider a thoughtful attribution process. Thus, too positive self-disclosure is more likely to be viewed as a form of disingenuous self-presentation that has ulterior motives. However, negative self-disclosure that is too extreme is also abnormal, considering that individuals tend to present themselves positively during initial interactions (Robinson et al., 1995).

In sum, previous studies have reported mixed and inconclusive findings. In this study, which we based on future cooperation, we adopted Miller et al. (2013) proposition that negative personal information is harmful to perceived trustworthiness because such information may reflect personal weakness. Moreover, disclosing dominantly positive information is normative on social media (Toma and Hancock, 2011); therefore, it may not be perceived that extremely positive information is an abnormal social cue. Thus, we proposed the following:

H2: Dominantly positive online self-disclosure attains a higher level of trustworthiness compared to neutral self-disclosure, followed by negative self-disclosure.

Trust is also considered an important component of interpersonal liking, and a higher level of trustworthiness perception leads to a higher level of interpersonal liking (Hawke and Heffernan, 2006). Trust plays a particularly important role when interacting with uncertain individuals, such as outgroup members or online strangers. Montoya and Pittinsky (2011) found that outgroup trust is positively associated with outgroup favoritism. Trust is key to the effect that group identification and relations have on outgroup liking, and it is difficult to form a “liking” between groups without trust, even if the groups highly identify with one another and are cooperative. Therefore, we predicted the following:

H3: A higher level of trustworthiness perception increases the level of perceived likability.

### Perceived Homophily as a Moderator

Perceived homophily is defined as “the degree to which pairs of individuals who interact are similar with respect to certain attributes, such as beliefs, values, education, social status, and the like” (Rogers and Bhowmik, 1970, p. 526). The similarity–trust/dissimilarity–distrust paradigm has been explained by social identity theory: individuals categorize others into “us” vs. “them” based on social categories, and they have a favorable perception (e.g., the trustworthiness perception) of members of the “us” group (Lau et al., 2008). Prisbell and Andersen (1980) advocated that perceived homophily could reduce uncertainty perception and positively affect feelings and safety perceptions in interpersonal interactions. Unlike in a group with prior interactions, where individuals can build knowledge-based trust, when interacting with online strangers, individuals form trust swiftly (e.g., ingroup trust) by using perceived homophily as a salient proxy/cue to reduce uncertainty (Robert et al., 2009).

In this study, when individuals viewed a stranger's social media profile, perceived homophily and information valence provided cues for judgments of perceived trustworthiness. Signaling theory suggests that individuals will make inferences based on any available data when they either do not have access to complete data or when they feel uncertain about the target person (Spence, 1974). However, when cues from a single aspect (e.g., valence) do not adequately reduce uncertainty regarding the trustworthiness perception of a stranger, how individuals derive more cues from other aspects (e.g., perceived homophily) is still uncertain. A previous study found that the effect of information valence on decision making was significant when a reader of an online travel site perceived a low level of similarity with a reviewer on the site (i.e., surface-level similarity) (Chan et al., 2017). A possible reason for this is that individuals tend to pay less attention to the information itself once they view the information source as being credible because of the perceived similarity, or vice versa. The relationship between the information itself and decision making therefore becomes either weaker or stronger, depending on the level of perceived similarity. Thus, we proposed the following hypothesis regarding the moderating effect of perceived homophily:

H4: Perceived homophily negatively moderates the effect of online self-disclosure valence on trustworthiness. More specifically, the influence of valence on trustworthiness will be stronger (weaker) when perceived homophily is low (high).

Overall, a combination of H1–H4 suggests a moderated mediation model. In other words, we predict that the valence of self-disclosure has a significant effect on perceived trustworthiness, which, in turn, affects interpersonal liking. The mediation effect of trustworthiness is influenced by perceived homophily. Thus, we propose the final hypothesis:

H5: The mediation effect of trustworthiness on the relationship between self-disclosure and likability is moderated by perceived homophily.

**Figure 1:**
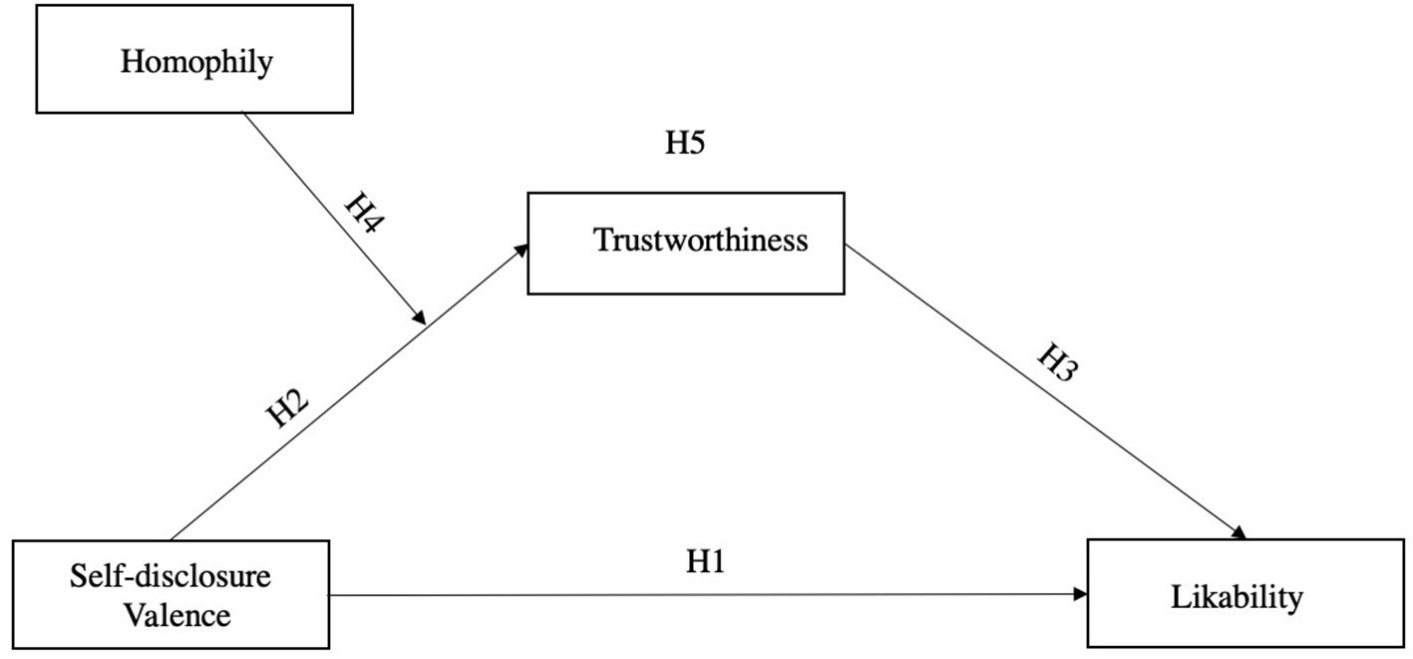
Conceptual model.

Figure 1 presents the oveall research model and hypotheses.

## Method

### Sample and Procedures

We collected data from October 6th to October 25th, 2018. We conducted two focus groups, two pretests, and an online experiment. The online experiment was conducted to examine the proposed hypotheses. To determine the sample size of the experiment, a power analysis was conducted using G^*^power 3.1 (Faul et al., 2009). Cohen (1988) suggested setting statistical power at 0.80 given an alpha level of 0.05, thus, at least 200 responses needed to be collected to detect a relatively small effect size (*b* = 0.25). A total of 224 undergraduates in China volunteered to participate in the study. After eliminating all incomplete answers, 204 valid questionnaires were used for data analysis. The participants' age was from 18 to 27 years old (*M* = 20.22; *SD* = 1.40), and 65.2% were female, and 34.8% were male. The recruited undergraduate students were sampled from various majors and universities to guarantee heterogeneity. A web link with access to the questionnaire was sent to each participant after they agreed to join the project. Each link, with one version of the stimuli, was randomly assigned by the online survey system. A consent form was provided at the beginning of the questionnaire, followed by a scenario introduction that read: “You have already accepted a friend request on WeChat from a stranger in your club. Your club leader told you that you would collaborate with him/her in the future, and now you are going to check his/her WeChat profile to get to know him/her.”

Each participant was randomly assigned to one of five experimental conditions that contained a mock-up of a WeChat profile that contained eight valenced posts by the “owner” of the profile (i.e., a mock-up collaborator). Each condition had different proportions of valenced self-disclosure posts generated by the collaborator that represented different levels of self-disclosure valence: (a) 100% positive self-disclosure posts; (b) 75% positive self-disclosure posts and 25% negative self-disclosure posts; (c) 50% positive self-disclosure posts and 50% negative self-disclosure posts; (d) 25% positive self-disclosure posts and 75% negative self-disclosure posts; and (e) 100% negative self-disclosure posts. To diminish the primacy effect and recency effect in impression formation, the valence of the first and last post was randomized in conditions (b), (c), and (d).

After reading their assigned profile, participants were asked to rate their perceived homophily, trustworthiness, likability, and valence. Participants also had to answer questions about their WeChat use and demographics. The experiment typically took less than 10 min.

### Stimuli and Manipulation

We chose WeChat profiles as stimuli since WeChat is one of the most popular social networking sites (SNSs) in China (Lin et al., 2017). Moreover, as noted earlier, the layout and content of WeChat profiles are more straightforward compared to other SNS profiles. This is beneficial as it minimizes any confounding factors in the experiment. The WeChat profile consists of five elements: the cover photo, profile owner's photo, profile owner's name, self-disclosure posts, and time of each self-disclosure message. For all stimuli, the cover photo, profile owner's photo, profile's owner's name, and time of each message were the same. We designed all of the stimuli to have no cover photo and used a neutral scenic photo as the profile owner's photo (see Supplementary Figure 1 for the actual stimuli).

The main purpose of this study is to estimate the effect of self-disclosure valence, which is conceptualized as the positivity, neutrality, or negativity of the information disclosed (Orben and Dunbar, 2017) and operationalized by the proportion of valenced posts in a social media profile. Therefore, we created five conditions based on the level of self-disclosure valence as noted in above.

To make the experiment stimuli more realistic and valid, as well as to better eliminate the confounding of self-disclosure topics and emotion types, we organized two online focus groups (each consisting of four to five undergraduates) to determine the appropriate topics (e.g., study, love, and interpersonal relationships) and emotion types for undergraduates' self-disclosure posts on WeChat. Drawn from the focus group's findings, 16 self-disclosure posts were created to reflect different valences of the designed profiles. Two pretests (*n* = 25, *n* = 5) were conducted to check the manipulation of the valence (positive vs. negative) of each self-disclosure message. The first pretest was performed to investigate the valence of 16 postings, and the second pretest was conducted to investigate the valence of five mock-up profiles in which these postings appeared. The results indicated that the manipulation in the current study was successful to the extent that participants could correctly distinguish between the valence of each posting and profile. Therefore, the five mock-up profiles were employed in the following actual experiment.

### Measures

For all the measures,^1^ we employed multiple item scales adapted from pre-validated studies. All items were translated into Chinese to ensure that participants could accurately understand the meaning of each item. Seven-point Likert scales were used throughout.

#### Likability

Likability was measured using an 8-item scale (α = 0.97) adapted from Reysen (2005). This scale is used to test the degree to which an individual is perceived as friendly and approachable (e.g., “This person is friendly,” “This person is warm,” “I would like to be friends with this person”).

#### Trustworthiness

The trustworthiness perception of the profile owner was assessed using seven semantic differential-type items (α = 0.90) adapted from the Individualized Trust Scale (Wheeless and Grotz, 1977). Items include “Trustworthy-Untrustworthy,” “Trustful of this person-Distrustful of this person,” “Confidential-Divulging,” “Candid-Deceptive,” “Not Deceitful-Deceitful,” “Straightforward-Tricky,” and “Honest-Dishonest.”

#### Homophily

Perceived homophily was assessed using three items (α = 0.95) adopted from the Perceived Homophily Measure (McCroskey et al., 1975) and comprised “The author thinks like me,” “The author behaves like me,” and “The author is similar to me.”

#### Control Variables

Similar to other relevant studies (e.g., Orben and Dunbar, 2017), this study also measured the participants' familiarity with WeChat, their intensity of WeChat use, and their demographics (e.g., age and gender) as controls, since these may influence the results. The familiarity of WeChat was tested by asking, “How long have you been actively using your WeChat account?” (*M* = 3.34, *SD* = 0.75). The intensity of WeChat use was assessed by two items: “On a typical day, how often do you check WeChat?” and “On a typical day, how often do you browse others' posts on WeChat?” (*M* = 3.95, *SD* = 1.16).

### Manipulation Checks

#### Perceived Valence

To test whether participants accurately perceived the dominant self-disclosure valence as we expected, we asked participants to report their level of agreement with “Most of the information is positive” on a 7-point Likert scale ranging from “1 = Strongly disagree” to “7 = Strongly agree.” The results of one-way ANOVA showed a significant difference between the five conditions [*F*_(4)_ = 34.09, *p* < 0.001], with the 100% positive conditions (*M* = 4.80, *SD* = 1.54) and 75% positive condition receiving higher scores (*M* = 4.75, *SD* = 1.75), followed by the neutral condition (*M* = 3.45, *SD* = 2.00), the 75% negative condition (*M* = 2.12, *SD* = 1.29), and the 100% negative condition (*M* = 1.58, *SD* = 1.22). The post hoc analysis showed that participants could distinguish between dominant positive, neutral, and dominant negative, while there was no significant difference between the 75% positive condition and the 100% positive condition (*M*_difference_ = −0.05, *p* = 0.89), as well as the 75% negative condition and the 100% negative condition (*M*_difference_ =0.54, *p* = 0.12). That is, as long as more than half of postings are positive/negative, the fine distinction (i.e., 100 vs. 75%) appeared to be unimportant. Thus, we combined the 100 and 75% groups to represent the dominantly positive valence condition and the dominantly negative valence condition, respectively. Following this, three conditions were determined (dominantly positive vs. neutral vs. dominantly negative).

## Results

The data were analyzed using IBM SPSS Statistics 22 and PROCESS macro 3.1 for SPSS. A One-Way ANOVA was employed to test our baseline hypothesis (H1), while Model 7 in SPSS PROCESS macro (Hayes, 2013) was employed to examine other hypotheses (H2-H5) involving mediation and moderation effects. We used 10,000 bias-corrected estimates and iterations. Online self-disclosure valence (IV) was operated as a multicategorical variable, and the negative condition was selected as the baseline group. Moderation effects of perceived homophily were investigated at plus (high level) and minus (low level) one standard deviation around the mean of perceived homophily.

**Figure 2:**
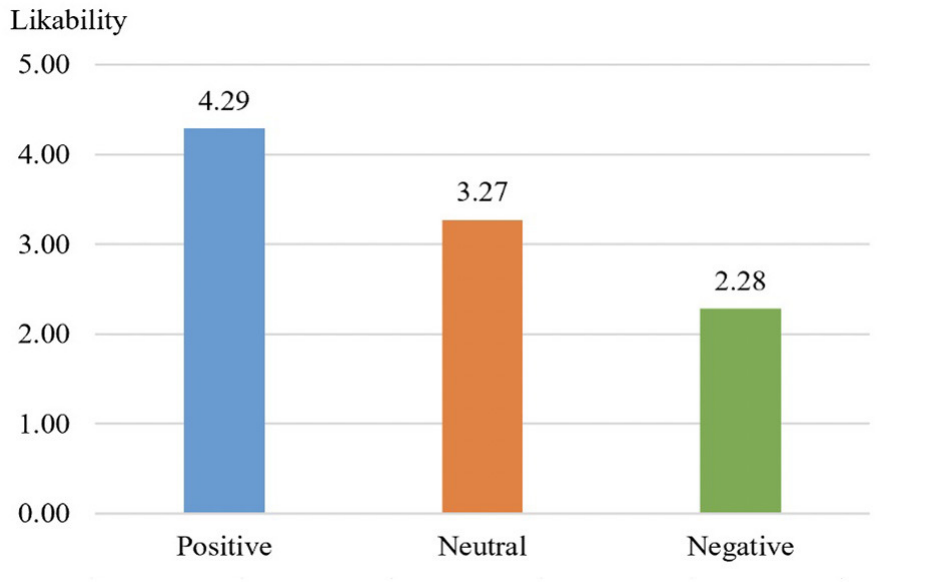
Mean differences between self-disclosure valances in likability.

H1 predicted the significant effect of self-disclosure valence on likability. Specifically, we predicted that dominantly positive self-disclosure would lead to the highest likability level, followed by neutral self-disclosure, followed in turn by dominantly negative self-disclosure. The result shown in Figure 2 indicated that there is a significant between-group difference in terms of likability [*F*_(2)_ = 44.43, *p* < 0.001]. Moreover, the profile owner with dominant positive self-disclosure information attained highest level of likability (*M* = 4.29, *SD* =1.27), followed by neutral self-disclosure (*M* = 3.27, *SD* = 1.56), and the least is dominant negative self-disclosure (*M* = 2.28, *SD* = 1.33). A Tukey's post hoc test was further conducted, and the result showed that there was a significant difference between each pair in terms of likability (positive vs. neutral: *p* < 0.001; positive vs., negative: *p* < 0.001; neutral vs. negative: *p* < 0.001), therefore, H1 was supported.

Table 1 presents the results of moderated mediation analyses, testing H2–H5. With regard to trustworthiness (H2), the result showed that dominantly positive self-disclosure acquired a significantly higher perception of trustworthiness compared to negative self-disclosure (*b* = 1.37, *p* < 0.001), while there was no significant difference between neutral self-disclosure and dominantly negative self-disclosure (*b* = 0.22, *p* = 0.59). Hence, H2 is partially supported.

**Table 1:** Moderated mediation effect of trustworthiness on likability.

**Effects on trustworthiness**		**Unstandardized *b***	**SE**	***t***	**95% CI**
Neutral	*(H2)*	0.22	0.42	0.54	−0.60 to 1.05
Positive		1.37^***^	0.38	3.63	0.63 to 2.12
Homophily		0.31^**^	0.12	2.61	0.08 to 0.55
Neutral^*^Homophily	*(H4)*	−0.00	0.15	−0.01	−0.30 to 0.30
Positive^*^Homophily		−0.30^*^	0.15	−2.01	−0.59 to −0.01
Familiarity of WeChat		−0.15	0.12	−1.25	−0.38 to 0.08
WeChat use intensity		−0.09	0.07	−1.19	−0.24 to 0.06
**Relatively direct effects on likability**					
Neutral		0.68^**^	0.22	3.07	0.24 to 1.11
Positive		1.50^***^	0.19	7.92	1.13 to 1.88
Trustworthiness *(H3)*		0.57^***^	0.07	8.70	0.44 to 0.70
Familiarity of Wechat		−0.26^*^	0.11	−2.37	−0.48 to −0.04
WeChat use intensity		0.08	0.07	1.16	−0.06 to 0.22
**Conditional indirect effects on likability (H5)**					
Neutral^*^Homophily		−0.00	0.15		−0.34 to 0.25
Neutral^*^Homophily (−1 SD)		0.13	0.17		−0.20 to 0.48
Neutral^*^Homophily (0 SD)		0.13	0.18		−0.26 to 0.42
Neutal^*^Homophily (+1 SD)		0.12	0.38		−0.73 to 0.73
Positive^*^Homophily		−0.17	0.16		−0.53 to 0.08
Positive^*^Homophily (−1 SD)		0.61	0.18		0.30 to 1.00
Positive^*^Homophily (0 SD)		0.37	0.17		0.02 to 0.67
Positive^*^Homophily (+1 SD)		0.10	0.38		−0.73 to 0.70

* *p <0.05*,

** *p <0.01*,

*** *p <0.001; Reference group is the negative condition, so the independent variables compare positive and neutral conditions with the negative condition; Regarding indirect effects, if zero is not included in the 95% confidence limits, the indirect effect test is significant; otherwise, it is non-significant*.

H3 predicted the direct positive effect of trustworthiness on likability. The result (see Table 1) showed that trustworthiness has a significant positive effect on likability (*b* = 0.57, *p* < 0.001). Thus, H3 is supported.

H4 predicted that perceived homophily moderated the effect of online self-disclosure valence on the perception of trustworthiness. The results showed (see Table 1) that perceived homophily had a significant interaction effect with dominantly positive self-disclosure on trust (*b* = −0.30, *p* < 0.05, *SE* = 0.15, 95% CI = −0.59 to −0.01). More specifically, the impact of dominantly positive self-disclosure on trustworthiness (operationalized here as the difference between positive self-disclosure and the baseline, negative self-disclosure) was significant when perceived homophily was at a low level (−1 SD) (*b* = 1.08, *p* < 0.001, *SE* = 0.27, 95% CI = 0.55 to 1.60) and at an average level (*b* = 0.65, *p* < 0.01, *SE* = 0.21, 95% CI = 0.23 to 1.07). In contrast, dominantly positive self-disclosure had no significant effect (*b* = 0.18, *p* = 0.61, *SE* = 0.35, 95% CI = −0.51 to 0.87) at a high level (+1 SD). This means that individuals rely on the self-disclosure valence to make an interpersonal judgment of trustworthiness when they interact with dissimilar individuals (i.e., low homophily), as they are likely to look for other signals to “fill the gap” and produce a trustworthiness perception due to increased levels of uncertainty associated with dissimilar others. However, the interaction between neutral self-disclosure and perceived homophily was nonsignificant (*b* = −0.00, *p* = 0.99, 95% CI = −0.30 to 0.30). Taken together with those of H2, the findings suggest that the difference in trustworthiness between neutral condition and negative condition was insignificant, regardless of perceived homophily. Thus, H4 is partially supported.

**Figure 3:**
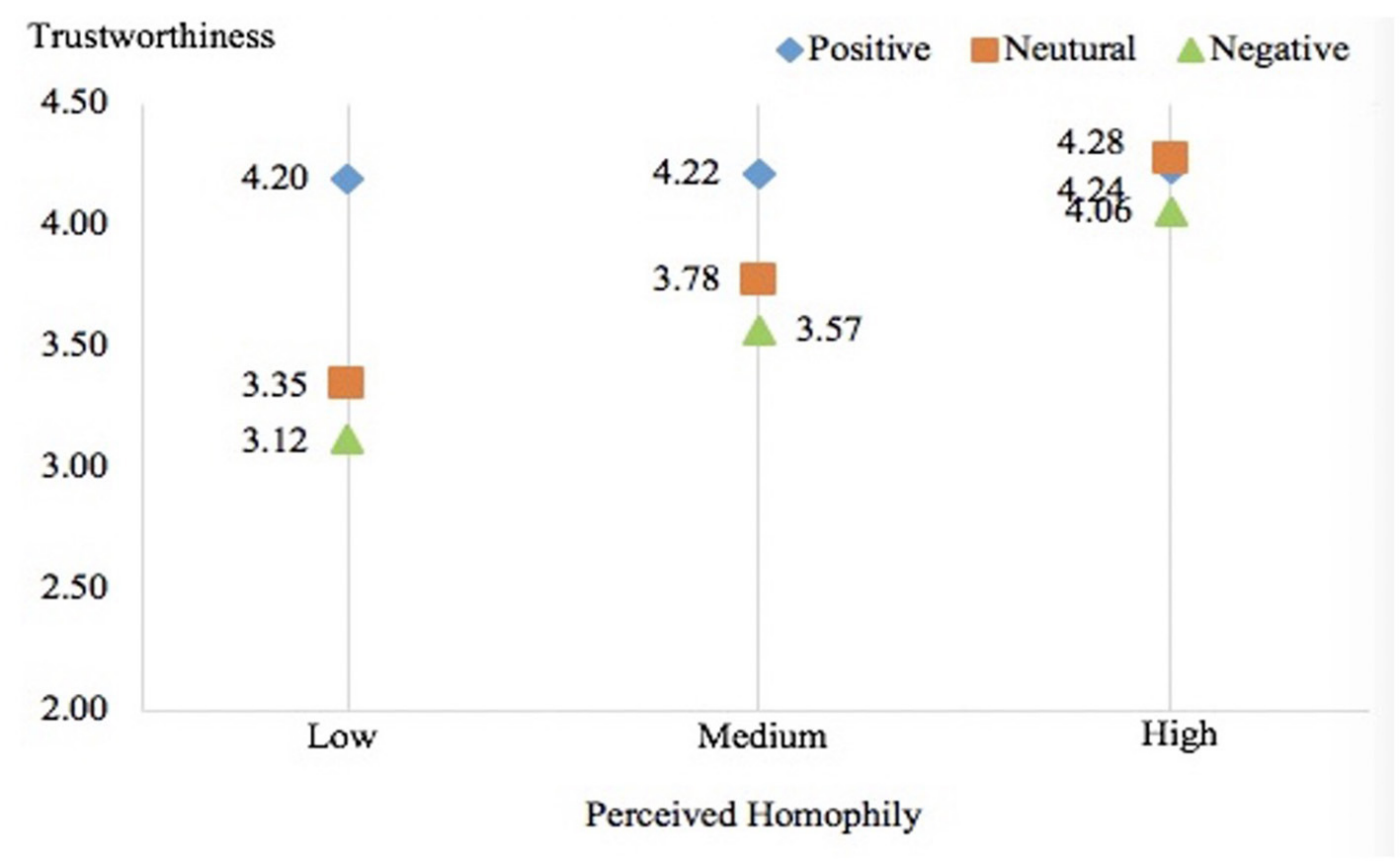
Patterns of interaction effects of valence and homophily on trustworthiness. This figure shows the estimated marginal means of trustworthniness at +1, 0, and 1 SD of perceived homophily.

Figure 3 shows estimated marginal means of trustworthiness across conditions, depicting overall interaction patterns. Although the positive condition attained the highest trustworthiness when perceived homophily is at a low or average level, the situation changed when perceived homophily is at high level as the neutral condition had the highest level of trustworthiness. Actually, the estimated marginal means of trustworthiness in three conditions were close to each other when perceived homophily was high, compared to the discrepancies in the three conditions when perceived homophily was at low or average level. It also indicated that the effect of self-disclosure valence on trustworthiness depends on the level of perceived homophily. In addition, Figure 3 shows the trustworthiness perception is relatively stable at different levels of perceived homophily; thus, the significant interaction effect founded in the positive condition may result from the difference between positive condition and negative condition (baseline).

It is worthwhile to note that, as shown in Figure 3, the neutral condition and the negative condition were almost identical (and substantially different from the positive condition), although the neutral condition consisted of the same proportion of positive posts and negative posts. The insignificant effects pertaining to the neutral condition observed in this study indicate that the negative self-disclosure posts seemed to weigh more than positive self-disclosure posts when the amount was the same.

H5 predicted that the mediation effect was moderated by perceived homophily. The results showed that compared to the negative condition (see Table 1), the moderated mediation effect in the positive condition depends on the level of perceived homophily. More specifically, the mediation effect of trustworthiness in the relationship between positive self-disclosure and likability was moderated by perceived homophily when perceived homophily was at a low level (−1 SD) (*b* = 0.61, 95% CI = 0.30 to 1.00) and at an average level (*b* = 0.37, 95% CI = 0.02 to 0.67), while a high level (+1 SD) of perceived homophily did not affect the mediation effect of trustworthiness (*b* = 0.10, 95% CI = −0.73 to 0.70). The relative direct effect of dominantly positive self-disclosure on likability (*b* direct = 1.50, *p* < 0.001, *SE* = 0.19, 95% CI = 1.13 to 1.88) remained significant, indicating that the effect of self-disclosure valence on likability was partially mediated by trustworthiness perception under positive conditions when the perceived homophily was at the lower or average level. However, although the direct effect of neutral self-disclosure on likability was still significant (*b* = 0.68, *p* < 0.01), there was no moderated mediation (*b* = −0.00, 95% CI = −0.34 to 0.25) across all neutral conditions as the path from the neutral valence to perceived trustworthiness was insignificant (*b* = 0.22, *p* = 0.59). Thus, H5 is partially supported.

## Discussion

The current study examined the effect of self-disclosure valence on first impression formation in the context of social media through online data from 204 WeChat users. This study also examined the mechanisms underlying this process by testing the mediation effect of trustworthiness and the moderation effect of perceived homophily. In sum, the results indicate that, although the valence of an impression judgment tends to be in line with a stranger's self-disclosure valence, the role of self-disclosure in the first impression is affected by the level of trustworthiness and perceived homophily. Trustworthiness plays an important role by mediating the effect of self-disclosure valence on likability. Perceived homophily moderates the mediation effect: individuals rely more on cues from self-disclosure valence when they feel dissimilar from strangers.

The findings discussed in the present study enrich the existing literature about online self-disclosure valence and first impression formation. First, the findings regarding the main effects that dominantly positive self-disclosure could have in terms of attaining the highest likability reinforce previous research, where it was found that positive self-disclosure has a positive influence on interpersonal perceptions, especially at the initial stages (e.g., Blau, 1964; Gilbert and Horenstein, 1975; Miller et al., 1992; Rains and Brunner, 2015). It confirms that self-disclosure valence is a central factor affecting interpersonal perceptions. For self-disclosure and interpersonal evaluation, it is usually positive self-disclosure that is linked with positive interpersonal evaluations, whereas negative self-disclosure is associated with negative interpersonal evaluations (Gilbert and Horenstein, 1975; Orben and Dunbar, 2017; i.e., the valence of interpersonal evaluation usually matches the valence of self-disclosure information). Miller et al. (1992) also found that individuals who positively disclose were judged as the most likable. Blau (1964) emphasized that individuals must present themselves in a positive way to obtain a favorable impression, especially in the early acquaintance stage. This is because an individual's negative self-disclosure in an initial encounter, comprising a display of deficiencies, would not succeed in conveying a signal that said individual is approachable (Blau, 1964).

Second, as for the relationship between self-disclosure valence and trustworthiness, the results are in line with previous studies that indicated positive self-disclosure is positively related to perceived trustworthiness (e.g., Runge and Archer, 1981; Miller et al., 2013). Previous studies have shown that negative or neutral self-disclosure leads to higher trustworthiness than positive self-disclosure does (e.g., Robinson et al., 1995; Ma et al., 2019). There could be a few reasons why disclosing dominantly positive information instead of dominantly negative information results in higher trustworthiness in this study. First, as discussed above, positive self-disclosure tends to produce a favorable impression. Thus, if individuals consider trustworthiness to be a good personal trait included in impression judgments, it is likely to be subjectively influenced by the overall impression instead of objectively affected by the credibility of self-disclosure information. In reality, individuals often have no access to others' self-disclosure information in terms of its “accuracy” (Miller et al., 1992); thus, perceived trustworthiness is likely to be considered as a subjective personal perception. Second, it might also be related to the different norms of self-disclosure on social media. Individuals are always motivated to selectively present themselves in CMC and disclose positive aspects of themselves to present an ideal self that has become prevalent on social media (Walther et al., 2015). Thus, disclosing dominantly positive information is normal and easy to accept.

Also, the findings show a positive relationship between trustworthiness and likability, which might have occurred because trustworthiness has a halo effect on first impressions, where limited information is given and there is a lack of previous interactions. The halo effect is formed through two different mechanisms: (a) individuals categorize overall impressions into either positive or negative evaluations that result in a single judgment, and (b) a single salient trait will be transferred onto an individual's judgments of other traits (Bierhoff and Klein, 1989). In the context of this study, we assume that both mechanisms may occur because some individuals may form a likability judgment, depending on their overall positive impression, which they could derive from perceived trustworthiness, while others may like the target because they consider trustworthiness a salient single trait. Previous literature has even emphasized that trust is the second most important antecedent of interpersonal liking, following personality (Hawke and Heffernan, 2006). Hence, perceived trustworthiness also plays an important mediating role in positive conditions.

As for the moderating role of perceived homophily, the results emphasized the role of different levels of perceived homophily which uncovered the different mechanisms in the course of making a trustworthiness judgment based on self-disclosure valence. The results showed that, when we encounter a stranger who is dissimilar or slightly similar to ourselves, we are more likely to rely on disclosure valence to form interpersonal trust. This is because, per uncertainty reduction theory, similarities can decrease uncertainty, whereas dissimilarities lead to increased uncertainty (Berger and Calabrese, 1975). The latter effect causes individuals to be more inclined to seek additional signals from relevant aspects (e.g., self-disclosure valence) to ensure that they make more accurate interpersonal judgments of strangers in a short period of time, according to signal theory (Spence, 1974). In contrast, when there are high levels of perceived homophily, individuals need not rely on other cues because they feel low levels of uncertainty. Moreover, the estimated marginal means of trustworthiness across six conditions showed that neutral conditions attained the highest trustworthiness when perceived homophily was high, which indicated that the effect of valence of trustworthiness does depend on the perceived homophily.

Consistent with the results of the moderation effect, dominantly positive self-disclosure has only a moderate mediation effect when perceived homophily is at the low/average level. An interaction pattern analysis indicates that, except when we encounter strangers who are quite similar to us, in most cases, we rely on self-disclosure valence to form our trustworthiness perception, which, in turn, has a positive effect on a first impression in terms of likability. Trustworthiness is important for helping us decide whether we like a stranger when we perceive a low/average level of homophily.

Lastly, the results related to the moderation effect and the moderated mediation effect suggest no significant difference between the neutral condition and the dominantly negative condition. This could be explained by negativity bias, which indicates that negative cues are more informative compared to positive cues in social cognition and interpersonal perception (Fiske, 1980). Individuals tend to hold a chronic positivity bias in interpersonal perceptions, where personal cues are predominantly positive and negative information is scarce, regardless of self-reporting or evaluations from others. Thus, while positive cues seem to apply to everyone, they are difficult to distinguish between, and they are universal and similar to modal cues that are defined as uninformative. In contrast, negative cues are highly valued for their rarity, which is in line with extremity effects (Fiske, 1980). Because negative self-disclosure cues embody more discriminant information, the levels of interpersonal liking and trustworthiness in the neutral condition are similar to those in the dominantly negative condition, regardless of perceived homophily, although the number of positive self-disclosure posts and negative self-disclosure posts are the same in the neutral condition.

Taken together, the findings in this study specify the different processes of first impression formation when individuals view different forms of valenced self-disclosure in strangers' online profiles. The most important theoretical implication is that the effect of self-disclosure valence on interpersonal perception is conditional, determined by relatively complex mechanisms involving mediation through trust and moderation by perceived homophily. The present findings thus not only offer a novel connection to a broader literature on interpersonal formation online but also inform understanding of when this psychological process is determined by self-disclosure valance, trust, and perceived homophily.

## Limitations and Future Study

There are a few limitations of the current study that may also provide directions for future studies. First, a clear limitation of our research is that it is impossible to completely separate emotion valence from the topics of the posts, as we adopted an experimental method. Similar to other studies that used experiments to estimate the effect of information valence, we conducted focus groups and pretests to find the optimal topics that would guarantee the realism and validity of the valenced information. Also, we intended to select the topics that undergraduates disclose in daily life, whether positive or negative, and ensured that these topics were balanced across the different conditions. However, we do not know to what degree these topics may contaminate interpersonal perceptions. For instance, perceivers who are single may have different attitudes toward positive/negative self-disclosures involving love (one of the topics in this study) compared to those of reviewers who are in relationships. We recommend that future experimental studies that deal with self-disclosure valence on WeChat focus on other topics or consider the interaction effect between the valence and the content so as to obtain more definitive results.

Secondly, the scenario that we established for the online experiment to ensure a realistic setting might have had an effect on the results. We believe that the participants' anticipation of future interaction encouraged them to deliberately make impression judgments of strangers, while at the same time, some participants may have paid more attention to “future cooperation” in the scenario and thus made task-oriented impression judgments. Hence, future research needs to minimize the scenario impact or create various scenarios to confirm these findings.

Third, though we explain the findings using motivational and cognitive approaches, we did not directly examine the role of related concepts, such as the level of uncertainty, motivations, and perceived risks, that are central to these explanations. Future studies should assess these variables and incorporate them in their research models to further verify the proposed theoretical mechanisms.

Last, although the Individualized Trust Scale used in this study is widely accepted in studies about self-disclosure and interpersonal trust, including recent research (e.g., Li et al., 2015; Hesse and Rauscher, 2019), the self-disclosure context is different from the context in which the scale was created decades ago. As a complicated concept, the judgment of trust could be influenced by the context, the task being evaluated, and the target person (Pascual-Ferrá, 2021). Thus, we suggest that future studies should employ or develop new comprehensive trust scales to test different social dimensions of trust.

## Conclusions

Valence is inherently embedded in self-disclosure, and it either unconsciously or consciously influences receivers' interpersonal perceptions. However, it is risky to consider the effects of valence on interpersonal perceptions without considering contexts and/or by neglecting the different mechanisms behind different valences. Thus, this study builds on the findings of previous studies on self-disclosure and contributes to this area of research by integrating trustworthiness as a mediator and perceived homophily as a moderator during first impression formation that is based on self-disclosure valence in social media profiles. The most interesting finding in our study was that perceived trustworthiness is essential to making a judgment of likability when we encounter strangers who are not highly similar to us. Trustworthiness formation occurs by seeking cues from both self-disclosure valence and perceived homophily. Also, negative self-disclosure is likely to have more discriminant power to influence trustworthiness perception compared to the same amount of positive self-disclosure. As noted earlier, we suggest that concerted efforts should be made to reveal the complex relationship between self-disclosure, valence, and impression formation online.

## Data Availability Statement

The raw data supporting the conclusions of this article will be made available by the authors, without undue reservation.

## Ethics Statement

The studies involving human participants were reviewed and approved by NUS Institutional Review Board (IRB). The patients/participants provided their written informed consent to participate in this study.

## Author Contributions

YQ was responsible for the entire research, including proposing the idea, collecting data, and writing. PL discussed the research with other authors all the time, analyzed the data, and tried to find a home for the paper. HC gave a lot of useful suggestions to design the study and improve the paper. LZ made an effort in data collection and paper writing. All authors contributed to the article and approved the submitted version.

## Conflict of Interest

The authors declare that the research was conducted in the absence of any commercial or financial relationships that could be construed as a potential conflict of interest.

## Footnotes

^1^We also measured perceived realism to check whether the experiment has ecological validity. However, the two items that created for this study failed to meet the reliability. Also, the perceived realism was not the focus of this study. Thus, the factor was not reported.
