# Supplementary figures and images for: First Impression Formation Based on Valenced Self-Disclosure in Social Media Profiles
Source: Front Psychol. 2021 Jun 18;12:656365. doi: 10.3389/fpsyg.2021.656365 (PMC8249806; doi:10.3389/fpsyg.2021.656365)

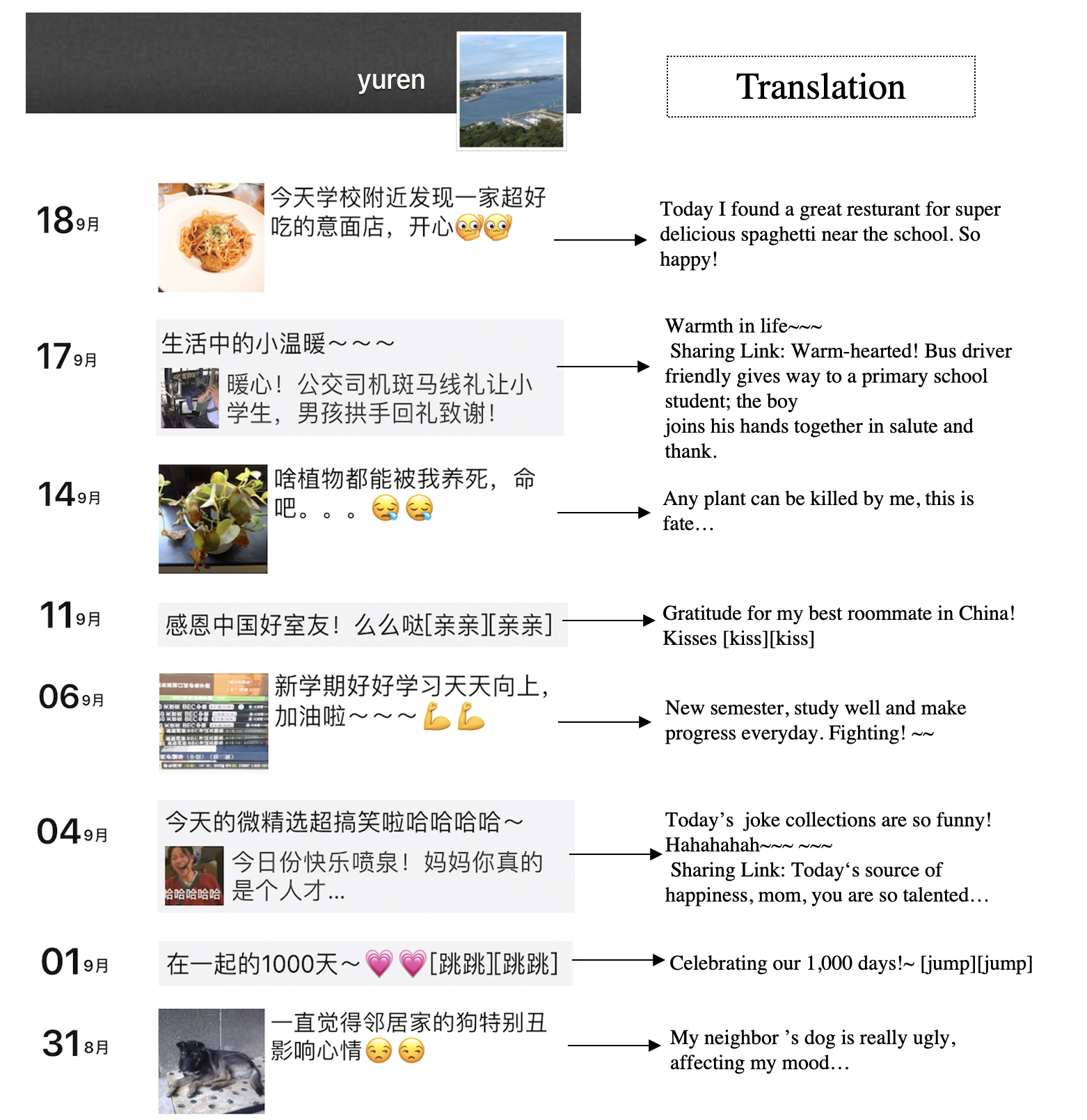

Supplement: Supplementary file 1 [file Image_1.png]
